# Advanced small-cell colon carcinoma: a case report

**DOI:** 10.1186/1752-1947-7-74

**Published:** 2013-03-18

**Authors:** Toshiaki Iwase, Yoshinori Masuda, Takayuki Suzuki, Osamu Takahashi, Masaru Miyazaki

**Affiliations:** 1 Division of Surgery, Heiwa Hospital, 29-1 Higashiterao Nakadai Tsurumi-ku, Yokohana city, Kanagawa, 230-0017, Japan; 2 Department of General Surgery, Chiba Graduate School of Medicine, 1-8-1 Inohana Chuo-ku, Chiba city, Chiba, 260-8677, Japan

**Keywords:** Chemotherapy, Small-cell carcinoma

## Abstract

**Introduction:**

Small-cell colon carcinoma is a very rare disease among colon neoplasms; it is difficult to achieve long-term survival due to its aggressive tumor behavior. Here we report the long-term survival of a patient with advanced small-cell colon carcinoma achieved by a combination of surgery and continuous chemotherapy.

**Case presentation:**

A 67-year-old Japanese man underwent abdominal computed tomography in our institution for follow up after gastrectomy, and abnormal thickness of the sigmoid colon wall was revealed. An endoscopy demonstrated a 20mm Bormann 2 lesion with central ulceration located 20cm from the anal verge. A sigmoidectomy was performed. Histologically, the tumor deeply invaded the tissue and extended beyond the serosa, and was diagnosed as small-cell carcinoma. Cisplatin plus irinotecan was administered for adjuvant chemotherapy. Nine months after surgery, a follow-up computed tomography showed an enlarged lymph node behind the inferior vena cava and a 15×8mm nodule located at the ventral side of the cecum. Under consideration of progressive disease, cisplatin plus irinotecan therapy was performed again using the same regimen. After nine cycles of cisplatin plus irinotecan therapy, a follow-up gastric endoscopy demonstrated external tumor invasion to the duodenum wall. Carboplatin plus etoposide therapy was selected as a third-line regimen. After six cycles of carboplatin plus etoposide therapy, the recurrence sites were maintained in a stable condition, and the survival time reached approximately 30 months after the initial surgery.

**Conclusions:**

We report the long-term survival of a patient with advanced small-cell colon carcinoma. In the future, the accumulation and analysis of rare cases that obtain a better survival time will contribute to clarifying neuroendocrine carcinoma biology, and help to improve the prognosis.

## Introduction

According to the classification of neuroendocrine tumors by the World Health Organization (WHO), small-cell colon carcinoma is categorized as a neuroendocrine carcinoma (NEC), which has high malignancy [1]. A NEC can occur anywhere in the gastrointestinal tract but it is rarely found in the colon: NEC has an incidence rate of only 0.6% of total colon malignancies [2]. Due to aggressive tumor behavior, distant metastases in internal organs or regional lymph nodes are often found at the same time as detection of a tumor. In addition to these facts, high recurrence rates contribute to poor survival, with a median survival of 10.5 months [2]. In patients with a metastatic lesion at the time of the surgery, the median survival time is only four months [3].

Here we report the long-term survival of a patient with advanced small-cell colon cancer who received continuous chemotherapy.

## Case presentation

A 67-year-old Japanese man underwent abdominal computed tomography (CT) in our institution for follow up after gastrectomy; the CT revealed abnormal thickness of his sigmoid colon wall and further studies were planned. Two years previously, he had undergone a distal gastrectomy due to gastric cancer (moderately differentiated adenocarcinoma), and received S-1 (tegafur-gimeracil, 100mg) orally for 14 days followed by a seven-day rest period as adjuvant therapy for two years. He also had inactive hepatitis C. There were no abnormalities on physical examination except for an upper abdominal midline scar due to previous surgery, and his performance status was grade 0. There were no abnormalities found on laboratory tests.

**Figure 1:**
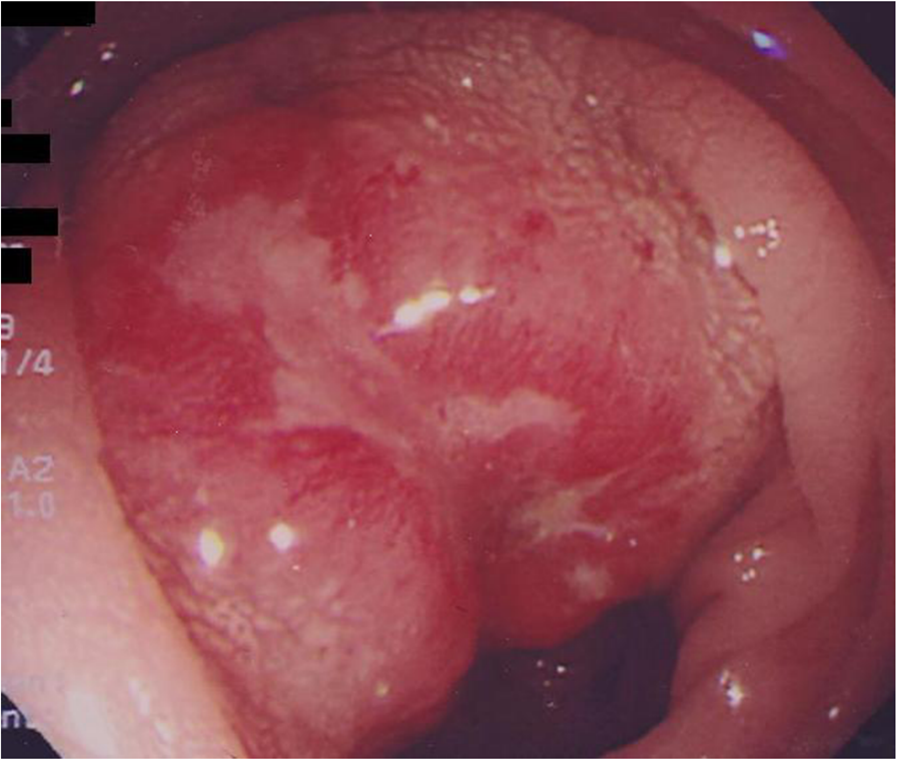
**Endoscopic findings.** An endoscopy demonstrated a 20mm Bormann 2 lesion with a central ulceration located 20cm from the anal verge.

A double contrast radiological examination with barium enema showed a protruding lesion with a smooth surface at the distal end of the sigmoid colon, and the lumen was narrowed for approximately 2cm. An endoscopy demonstrated a 20mm Bormann 2 lesion with central ulceration located 20cm from the anal verge (Figure 1). No metastatic sites in other internal organs were confirmed by CT.

Multiple biopsies from the main site showed group V malignant cells that were positive for chromogranin A, synaptophysin, and CD56, and proved to be a NEC.

Under a diagnosis of NEC, a sigmoidectomy was performed. During the operation, three small hard white nodes were found at the mesenteric and pelvic cavity. The main tumor was located at 20cm on the oral side of the peritoneal reflection, and dissected by three enlarged paracolic lymph nodes.

**Figure 2:**
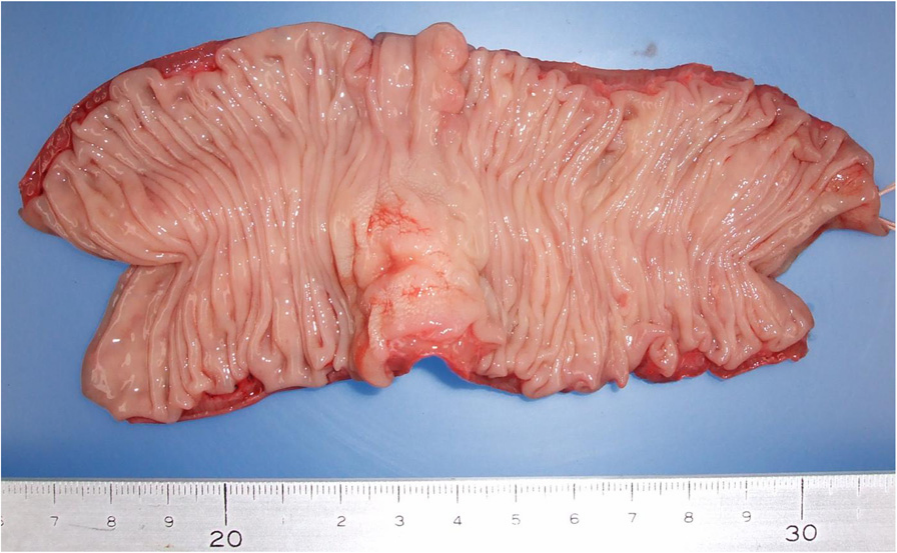
**Macroscopic findings.** A 15×20mm sub-mucosal tumor with the appearance of a small central ulceration was observed in the sigmoid colon.

Macroscopically, a 15×20mm sub-mucosal tumor with the appearance of a small central ulceration was observed in the sigmoid colon (Figure 2).

**Figure 3:**
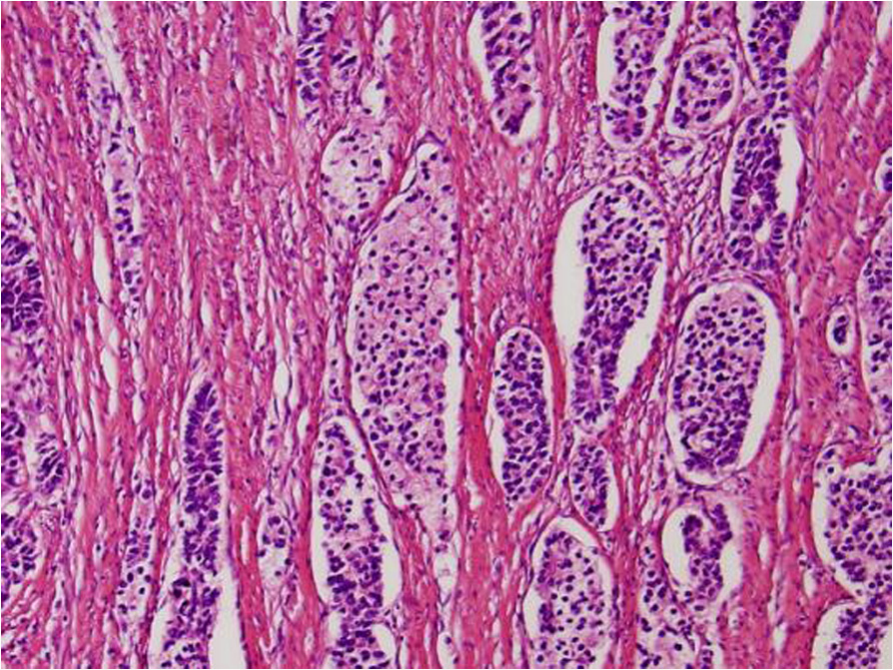
Pathological appearance of small-cell colon carcinoma on hematoxylin and eosin staining (original magnification ×100).

**Figure 4:**
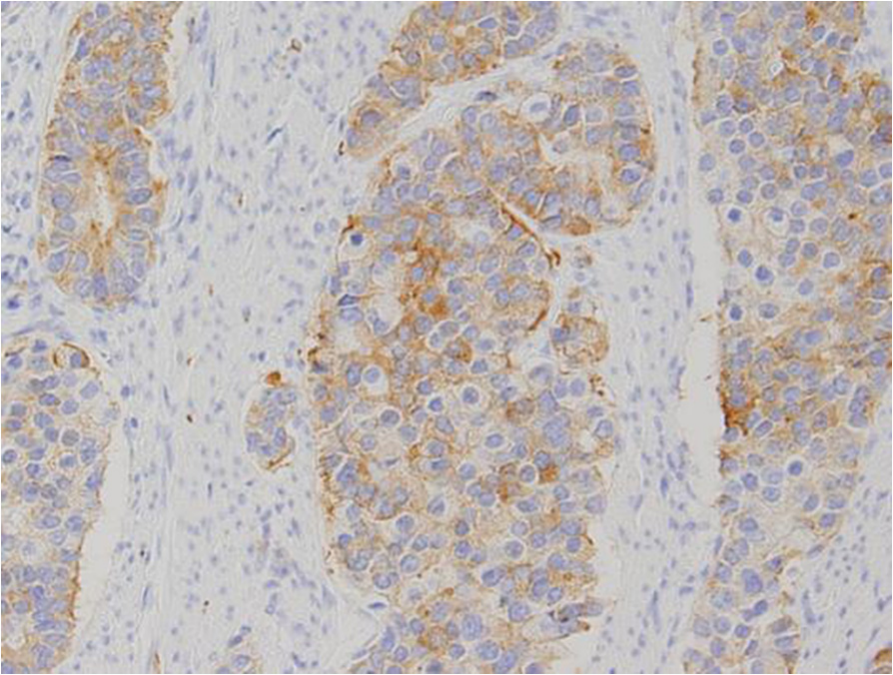
Synaptophysin was positive on immunohistochemical staining (original magnification ×100).

**Figure 5:**
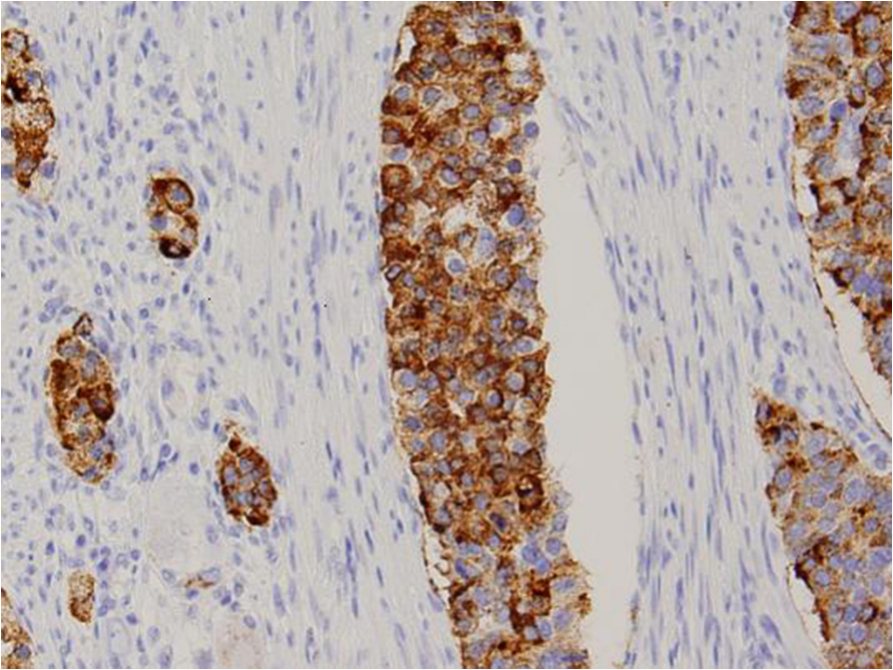
Chromogranin A was positive on immunohistochemical staining (original magnification ×100).

Microscopically, the tumor deeply invaded the tissue and extended beyond the serosa. On hematoxylin and eosin staining, the tumor had high cellularity with appearance of hyperchromatic nuclei with irregular sheets, rosette arrangements, and alveolar structures (Figure 3). Immunohistochemical staining for chromogranin A, synaptophysin, and CD56 were also positive, which indicates an endocrine function (Figures 4 and 5). Several ductal structures were occupied by mucinous fluid and positivity for periodic acid-Schiff (PAS), PAS-diastase (d-PAS), and Alcian blue was observed on the mucosal surface, suggesting the adenocarcinoma was included within the tumor. The nodules in the mesenteric and paracolic lymph nodes were also pathologically diagnosed as metastases. Finally, the tumor was diagnosed as a NEC (small-cell carcinoma).

Adjuvant chemotherapy was planned based on a regimen for small-cell lung carcinoma. Cisplatin plus irinotecan (cisplatin plus irinotecan therapy, cisplatin 60mg/m^2^ day 1 and irinotecan 60mg/m^2^ days 1, 8, and 15) were administered. Only a grade 2 side effect of transaminitis was observed during chemotherapy, and the patient recovered conservatively from this side effect. After finishing five cycles of chemotherapy, an evaluation CT showed no findings of recurrence. After cisplatin plus irinotecan therapy was finished, tegafur-uracil (UFT; 400mg) and leucovorin (UZEL, 75mg) were administered orally for 28 days followed by a seven-day rest period. Nine months after surgery, follow-up CT showed an enlarged lymph node behind the inferior vena cava and a 15×8mm nodule located at the ventral side of the cecum, which raised a suspicion of mesenteric dissemination. Under consideration of progressive disease, cisplatin plus irinotecan therapy was performed again using the same regimen. After nine cycles of cisplatin plus irinotecan therapy, a follow-up gastric endoscopy demonstrated external tumor invasion to the anterior duodenum wall due to growing peritoneal dissemination. It seemed that the tumor had acquired a resistance to cisplatin plus irinotecan therapy, and therefore carboplatin plus etoposide therapy, carboplatin plus etoposide therapy carboplatin 390mg day 1 + etoposide 96mg, 80% dose, days 1, 2, 3 were selected as a third-line regimen. After six cycles of carboplatin plus etoposide therapy, the peritoneal dissemination sites had not expanded on CT findings, and the tumor invasion on the anterior duodenum wall had partially receded on endoscopic findings. At the present moment, the survival time has reached approximately 30 months after the initial surgery.

## Discussion

According to the 2010 WHO classification of neuroendocrine neoplasia (NET), NET was categorized into grade 1 and grade 2 based on the malignant potential of metastasis, and tumors with high malignancy were divided into NEC or mixed adenoendocrine carcinoma, which includes an element of adenocarcinoma of more than 30% [1]. The present case was pathologically diagnosed as a small-cell carcinoma mixed with poor to moderate adenocarcinoma elements of less than 30%, and was categorized as NEC based on WHO classifications. When applying the present WHO classification, special attention should be paid because previously well or poorly differentiated endocrine carcinomas are redefined separately as NET grade 2 and NEC.

A definitive diagnosis was made histopathologically, and immunohistochemical staining greatly helps in diagnosis [4]. Saclarides *et al*. reported that more than a few cases initially diagnosed as carcinoid were changed to NEC after histopathological analysis [3]. In the present case, diagnosis was made preoperatively based on the immunohistochemical staining of chromogranin A, synaptophysin, and CD56. An endoscopic biopsy diagnosis is occasionally changed from well to moderately differentiated colon adenocarcinoma to NEC by pathological evaluations after surgery [3]. When any specific structures of NEC are histologically found, routine immunohistochemical staining contributes to appropriate diagnosis and continuous early treatment.

Chemotherapy for NEC is often performed based on the regimen for small-cell lung carcinoma. The latest guidelines for small-cell lung carcinoma from the National Comprehensive Cancer Network (NCCN) recommend etoposide plus platinum agents such as cisplatin or carboplatin for limited stage, and adding irinotecan instead of etoposide for extensive stage [5]. The present case initially received cisplatin plus irinotecan therapy, and secondly carboplatin plus etoposide therapy was selected because the tumor acquired resistance to cisplatin plus irinotecan therapy. In the present case, which was considered to have a high recurrence risk, UFT and UZEL were selected for maintenance and carboplatin plus etoposide therapy after the first cisplatin plus irinotecan therapy was administered based on a colon cancer regimen. Although the disease-free interval from the first cisplatin plus irinotecan therapy to recurrence was approximately four months, cisplatin plus irinotecan therapy was selected again. In the NCCN guidelines for small-cell lung cancer, when a recurrence is observed less than six months after finishing chemotherapy, a different treatment regimen is recommended [5]. It might have been better to change from cisplatin plus irinotecan therapy to carboplatin plus etoposide therapy at the time of recurrence because the tumor had already shown resistance to cisplatin plus irinotecan therapy.

It is very rare for patients with small-cell colon carcinoma to survive for approximately 30 months after being diagnosed with stage IV disease and peritoneal disseminations. Some reports have shown long-term survival despite an advanced stage by combination of operation and effective chemotherapy [6,7]. Unfortunately, patient characteristics associated with positive results from chemotherapy and favorable outcomes are still unknown because so few cases achieve long-term survival. A recent clinical trial showed the efficacy of amrubicin, an anthracycline agent with potent topoisomerase II inhibition activity, in improving survival time as second-line treatment for patients with small-cell lung cancer [8]. Although the number of cases was limited, Asayama *et al*. also reported the efficacy of amrubicin as first-line treatment for five cases of gastrointestinal endocrine carcinoma [9]. Earlier reports suggested that a certain percentage of patients with small-cell colon carcinoma had sensitivity for chemotherapy and achieved a better prognosis [6,7].

In general, small-cell colon carcinoma has a high risk for recurrence and the follow-up method is important. Because recurrence sites are found in various organs, such as lymph nodes, liver, lung, and brain, less invasive whole body screening is needed. A recent study showed the effectiveness of somatostatin receptor scintigraphy on diagnosis and follow up by using an ^111^indium pentetreotide that specifically associated with the somatostatin receptor in NEC [10]. However, the superiority of CT, magnetic resonance imaging, and somatostatin receptor scintigraphy is still controversial, and regular whole body screening and reassessment of chemotherapeutic effect is essential.

## Conclusion

In this patient, long-term survival was achieved with a combination of surgery and chemotherapy, even though the cancer was at an advanced stage. The ideal strategy for advanced small-cell colon carcinoma has not yet been established. However, if it is determined before the operation that the tumor is advanced, then tumor resection should be performed, and selecting a chemotherapy regimen based on established guidelines will contribute to a better prognosis.

In the future, the accumulation and analysis of rare cases that obtain a better survival time will contribute to clarify NEC biology, and help to improve the prognosis.

## Consent

Written informed consent was obtained from the patient for publication of this case report and accompanying images. A copy of the written consent is available for review by the Editor-in-Chief of this journal.

## Competing interests

The authors declare that they have no competing interests.

## Authors’ contributions

TI: acquired the data and composed the manuscript. YM: interpretation and manuscript revision. TS: interpretation and manuscript revision. OT: interpretation and manuscript revision. MM: interpretation and manuscript revision. All authors read and approved the final manuscript.
